# The Impact of Pharmaceutical Home Care on Medical Utilization for Frequent Users of Outpatient Services in Taiwan

**DOI:** 10.3390/ijerph18147336

**Published:** 2021-07-08

**Authors:** Chien-Ying Lee, Heng-Hsuan Su, Yu-Chia Chang, Tung-Han Tsai, Yung-Rung Lai, Kuang-Hua Huang

**Affiliations:** 1 Department of Pharmacy, Chung Shan Medical University Hospital, Taichung 40201, Taiwan; cshd015@csmu.edu.tw (C.-Y.L.); cshd050@gmail.com (Y.-R.L.); 2 Department of Pharmacology, Chung Shan Medical University, Taichung 40201, Taiwan; 3 National Health Insurance Administration, Ministry of Health and Welfare, Taipei 106211, Taiwan; judy881133@gmail.com; 4 Department of Health Services Administration, China Medical University, Taichung 40402, Taiwan; dondon0525@gmail.com; 5 Department of Healthcare Administration, Asia University, Taichung 41354, Taiwan; ycchang@asia.edu.tw; 6 Department of Medical Research, China Medical University Hospital, China Medical University, Taichung 40402, Taiwan

**Keywords:** pharmaceutical care, utilization review, ambulatory care, primary healthcare

## Abstract

Due to the high-accessibility and low-copayment of healthcare system in Taiwan, the clinical visit frequency of people is relatively high, which often leads to an excessively high healthcare expenditure. The aim of this research was to explore the effectiveness of pharmaceutical home care for frequent users of outpatient service and to analyze the impact of pharmaceutical home care on medical utilization. The study was based on the Taiwan National Health Insurance Research Database. Patients with over 100 clinical visits during 2010 to 2012 were selected as subjects. Whether these patients participate the experimental plan of pharmaceutical home care in the following year and the medical utilization of the research subjects were analyzed to compare the difference between participating group and non-participating group in this plan. The generalized estimating equation was employed to examine the difference of medical utilization. A total of 3943 subjects were included in this study, including 591 patients (14.99%) participating in the experimental plan. The average number of physician visits during the following year of the participating group was higher than that of the non-participating group by 0.12 visits, and the outpatient medical expense was lower than the non-participating group by 18,302 points (1 point = 0.03 US dollars). After participating in the plan, the average number of clinical visits of frequent users of outpatient services was significantly reduced by 6.63 visits, and the outpatient expense was significantly decreased by 9871 points. After joining the experimental plan of pharmaceutical home care, the average number of outpatient visits decreased significantly and the medical expense was lower when compared with those who did not participate in the plan.

## 1. Introduction

In Taiwan, the National Health Insurance (NHI) program was introduced in 1995 [1]. The NHI system provides medical insurance coverage for people, with nearly the whole of Taiwan’s population having been covered with comprehensive insurance [2]. NHI was associated in a reduction in mortality rate and considered to be amenable to health care, particularly among those uninsured people less likely to have medical insurance previously [2]. Literatures have different definitions of patients with high frequency visits. A Canadian study by Demers defined a high use of ambulatory care on patients who received care from more than 20 physicians annually, and received 10 times more medical services than the overall population [3]. Hauswaldt et al. studied chronically ill and multimorbid patients and defined frequent attenders as 24 or more contacts per year [4]. Another study for the medical utilization of the elderly over 65 years old has established a criteria for frequent attendance with ≥50 contacts with practices, contacts with ≥10 different individual practices, or ≥3 practices of the same discipline per year. Anyone who meets one of the three criteria is defined as a high frequency attendance [5].

The healthcare system in Taiwan is characterized by good accessibility, comprehensive population coverage, and low-copayment, which has contributed to a tendency of people seeking medical service for not feeling well [6]. Patients with multiple chronic conditions (MCCs) tends to be highly frequent users of medical resources. Among all age groups, the percentage of MCC patients are increasing to affect various aspects in healthcare [7] since MCC patients were associated with an excessively high healthcare expenditure [8].

Frequent visits have always been an issue for health insurance. The superiority of the single-payer systems is evidenced by the performance of the Taiwanese health care system [9]. The high accessibility, comprehensive population coverage, and low-copayment condition of the Taiwanese healthcare system have led to a doctor-shopping behavior of people [6,10]. These doctor-shopping patients often spend an excessively high healthcare expenditure [8]. In order to rationalize the use of medical resources, the NHI Administration has implemented the NHI High-visit Medical Behavior Improvement Program—Pharmaceutical Home Care Pilot Program for patients with frequent visits since 2010. Patients with a number of outpatient visits ≥100 were eligible for the Pharmaceutical Home Care Pilot Program. Based on the consideration of the disease severity of patients and manpower of community pharmacies, the priorities of participating in the program were as follows. First, patients had two or more chronic diseases and have received more than 13 chronic disease prescription from two or more hospitals; Second, medicine expenses were in the top 50%, and the number of hospitals was more than 6; Third, Half of the prescriptions and medicines were more than 6; Fourth, physicians or health insurance agency think there was a need for pharmacists.

The aims of this research are to analyze the characteristics of frequent users of outpatient services, to explore the effectiveness of frequent users of outpatient services participating in the experimental plan of pharmaceutical home care, and to analyze the impact of pharmaceutical home care on medical utilization for frequent users of outpatient services.

## 2. Materials and Methods

### 2.1. Data Source

This study was a secondary analysis. Data were obtained from the 2010–2013 Longitudinal Health Insurance Database (LHID) provided by the National Health Insurance Administration, Ministry of Health and Welfare Taiwan. The LHID contained one million beneficiaries, which stratified random sampling by gender, age, and township from all insured of National health insurance Taiwan (NHI). The NHI covering over 99% population in Taiwan, avoiding bias from selection, non-response, or poor recall. The LHID has been shown to have good levels of accuracy and completeness in recording prescriptions and clinical diagnoses. Owing to the anonymity of the database, the requirement for informed consent was waived, and this study was approved as an ethical review by the Institutional Review Board of China Medical University Hospital, Taiwan (Approval date: 21 September 2018, No: CMUH106-REC1-134).

### 2.2. Study Subjects

All study subjects were patients with a high frequency of visits between 2010 and 2012, with a number of outpatient visits ≥100 in the current year, whereas not all patients with a high frequency of visits were mandatory to enroll in the program in the following year. Therefore, the study further divided into groups of participating or non-participating the Pharmaceutical Home Care Pilot Program in the following year based on the health insurance declaration record. The status of participating in the program is defined as the prescription and dispensing details file in NHIRD and the declared case is classified as the Pharmaceutical Home Care Pilot Program.

### 2.3. Study Design

This research is a cross-sectional study by controlling related variables to explore the impact of Pharmaceutical Home Care Pilot Program on the medical utilization. Every patient was only included once to avoid duplicate observation. Whether these patients participate in the pharmaceutical home care in the following year, and the medical utilization of the research subjects, aided in analyzing the impact of participation on medical utilization.

Controlled variables include patient characteristics (age, gender, degree of urbanization, and Charlson Comorbidity Index (CCI), comorbid conditions (hypertension, diabetes, allergic rhinitis, musculoskeletal system and connective tissue diseases, digestive system diseases, sleep disturbances, and dizziness). The dependent variables are the number of outpatient visits and outpatient medical expenses in the current year. The unit of medical expense claim in the LHID was the “point”. One point was approximately equal to 0.03 US dollars.

### 2.4. Statistical Analysis

SAS 9.4 software was used for data sorting and statistical analysis with a *p* value < 0.05 as statistical significance. First of all, we used descriptive statistics to understand the basic characteristics of subjects, and then t-test and ANOVA were conducted for double-variable analysis of participation in the pharmaceutical home care pilot program and medical utilization. Because the distribution of the healthcare expenditure was markedly skewed, we used the generalized estimating equation (GEE) to analyze the impact of the pharmaceutical home care pilot program on the medical utilization after being adjusted for the variables, as otherwise the estimation results would have been biased.

## 3. Results

### 3.1. The Baseline Characteristics of Patients

The basic characteristics of subjects are shown in Table 1. A total of 3943 frequent users of outpatient services were eligible to be included in the pharmaceutical home care pilot program. Eventually, there were 591 (14.99%) patients participating. The participation rate is higher for female subjects (15.15%) compared to that of male subjects (14.84%).

Regarding to basic characteristics of subjects, male, aged between 75 and 85, high degree urbanization and a CCI score of 1 to 2 were accounted for a higher percentage of subjects. Regarding comorbidities, there were 3318 (84.15%) patients seeking medical care for digestive diseases, 3293 (83.52%) patients for musculoskeletal system and connective tissue diseases, and 2524 (64.01%) patients for hypertensive diseases.

### 3.2. Influencing Factors for the Willingness of Patients with Frequent Visits to Participate the Plan

Table 2 indicated the factors relevant to the participation of patients with frequent visits into the plan. Based on the GEE model analysis with female subjects as the reference group, the odds ratio of men joining the project is 1.06 times (95% CI = 0.86–1.30), without reaching a statistical significance. In the aspect of age, with subjects under 44 years old as the reference, the age groups of 55–64 years (OR = 1.45, 95% CI = 0.90–2.31) and 65–74 years old (OR = 1.41, 95% CI = 0.89–2.23) have higher participate rates, while the age group of over 85 years old (OR = 0.69, 95% CI = 0.36–1.31) has the lowest rate, however, the difference is not statistically significant. Regarding the degree of urbanization with the high urbanization as the reference group, the odds ratio of participation is 1.41 for patients in the middle urbanization (95% CI = 1.11–1.80) and 1.66 for patients in low-urbanization areas are (95% CI = 1.26–2.19). In the aspect of CCI scores with a CCI score of 0 as the reference group, the odds ratio of participation for patients with the CCI score of 1 to 2 was 1.42 times (95% CI = 0.95–2.11) without statistical significance. As for comorbidities, the odds ratio of participation is 1.30 for hypertension (95% CI = 1.04–1.62, *p* = 0.022), and 1.26 for vertigo (95% CI = 1.04–1.54, *p* = 0.021), and both reached a statistically significant difference.

### 3.3. The Impact of Participation on the Frequency of Outpatient Medical Utilization

GEE model was used to analyze the difference between two subject groups in the number of outpatient visit. Results shown in Table 3 revealed that the average number of outpatient visits was 105.35 and 92.78 for patients participating and non-participating in the plan, respectively. After controlling related variables, the average number of outpatient visits for those who joined the plan was 0.117 higher than those who did not join the plan (95%CI = 0.08–0.15, *p* < 0.001). With the group under 44 years old as reference, the participation rate of patients over 85 years was the lowest, with 0.118 visits lower than the reference group (95% CI = −0.20 to −0.03, *p* = 0.007). Using the high degree of urbanization as the reference group, the average number of outpatient visits for the middle is 0.048 visits lower (95% CI = −0.09 to −0.01, *p* = 0.018). As for comorbidities, the average number of outpatient visits for patients with musculoskeletal system diseases and connective tissue diseases was 0.121 visits higher than those of without such comorbidities (95% CI = 0.08–0.17, *p* < 0.001), and for patients with digestive system diseases, it was 0.042 visits higher than those without such comorbidities (95% CI = 0.00–0.08, *p* = 0.038). The average number of visits for patients with dizziness was 0.032 visits higher than that of patients without such comorbidities (95% CI = 0.00–0.06, *p* = 0.048).

### 3.4. The Impact of Pharmaceutical Home Care Plan on Outpatient Medical Expenses

Results shown in Table 4 were the difference in outpatient medical expenses between participating and non-participating groups. 

The results indicated that the average outpatient medical expense points of patients in the participating group was 81,269 points less than that of non-participating group (95% CI = −26,301 to −10,303; *p* < 0.001). As for gender, the average outpatient medical cost of male subjects was 6738 points more, but not significantly, than that of female subjects (95% CI = −10,749 to 14,945; *p* = 0.749). As compared with subjects of the high degree of urbanization, the average outpatient medical expenses of subjects with middle (89,278 points) and low degrees (83,720 points) of urbanization had a statistically significant decrease. Regarding the effect of CCI, a higher CCI score was significantly related to a higher average of outpatient medical expenses. For the impact of comorbidities, the average outpatient medical expenses of patients with dizziness is 87,866 points less than those without such comorbidities (95% CI = −27,862 to −8228; *p* < 0.001), while there was no statistically significant difference for other comorbidities.

### 3.5. Differences in Medical Utilization before and after the Participation in the Plan

The paired t-test was used to analyze the average number of outpatient visits and the average medical expenses before and after the participation of subjects among the patients joining the pharmaceutical home care plan, as shown in Table 5. Results showed that after participating in the plan, the average number of outpatient visits decreased for 6.63 visits and the average outpatient cost significantly decreased by 9871 points.

## 4. Discussion

Pharmaceutical care has evolved from passively waiting for patients in the past and has now developed into an active provision of services, and community pharmaceutical care has also become a trend [11]. Several positive outcomes obtained with different pharmaceutical care programs are making a beneficial change based on the pharmacist’s professional judgment by applying continuous quality improvement [12]. This plan is the first time that a community pharmacist provides pharmaceutical care for patients with frequent visits. This study tried to evaluate the influencing factors of patients with frequent visits in the pharmaceutical home care program, and the impact of the plan on the medical utilization of frequent users.

In this study, a total of 3943 people were identified as having visited physicians for over 100 times. The percentage of these patients participating in the experimental plan of pharmaceutical home care was 14.99%. The influencing factors for these subjects participating in the plan include urbanization level, hypertension, and dizziness. The average physician visits during the following year for participating group was higher than non-participating group for 0.117 visits, and the outpatient medical expenses was lower than the non-participating group by 18,302 points. After participating in the plan, the physician visits of studied subjects significantly reduced by 6.63 visits, and the outpatient expense was significantly decreased for 9871 points. The influencing factors for the number of physician visits were age, degree of urbanization level, and other comorbidities, including musculoskeletal system diseases, connective tissue diseases, digestive system disease, and vertigo. The influencing factors for outpatient medical expenses in the following year include degree of urbanization level, CCI index, and comorbidity dizziness. There were no significant differences in the influence of pharmacist characteristics on the medical utilization for outpatients who joined the experimental plan.

High utilizers are associated with a higher proportion of medical services and therefore produce relative health care services expenditures [13,14]. Several studies showed that chronic diseases and multi-morbidity are associated with substantially higher health care utilization [4,15]. The frequent visitors has some common characteristics. A study found that most over-users are elderly, female, less-educated, self-perceived patients with poor health and multiple clinical treatments [16]. A German study showed that patients with frequent visits are mainly elderly, suffering from severe or multiple diseases, and relying on long-term care [5]. A study from US Medicare files expressed that patients with medical expenses in the top 10% are distributed among older male, more often black, and more co-morbidity [17]. In addition, a Danish study found that patients with hypertension, diabetes mellitus, and mental illness have twice the odds of becoming a patient with high frequency visits [18].

In this study, we found that the characteristics of outpatients who visited more than 100 times were mainly male, aged from 65 to 85 years old, and high urbanization. The influencing factors of becoming a patient with frequent visits include female, aged over 85 years old and higher total number of medications [19]. Research in South Korea found that most of the overuse of medical resources was from those who were elderly and female [16]. These factors were similar to that identified from other literature research, while the gender distribution is slightly different. It is speculated that it may be different due to different national conditions. Taiwan’s medical utility is mainly concentrated in areas with high urbanization. The scholars use the degree of urbanization developed by Liu et al. to explore the differences in medical use between different degrees of urbanization in Taiwan and find that the degree of urbanization is grade sixth agricultural towns have a significantly lower medical utilization than grade 1 highly urbanized towns [1].

In this study, most of the patients had CCI scores of 1–2. Canadian scholars found that the average CCI index of the high-visiting group with an age of over 65 was 5.5 points, which is significantly higher than the average for the general group of 4.1 points [19]. The distribution of the CCI score was slightly different in other studies. A literature pointed out that patients with significant psychiatric and social comorbidities was prone to high frequency visits [20]. Several studies indicated that there is an association between high utilization and psychiatric syndromes, and medically unexplained symptoms [21,22]. The medical home care pilot program excluded the number of consultations in the psychiatric department. Therefore, this may be one explanation for different CCI score in this study. The influencing comorbidities for frequent users of outpatient services who participated the plan were hypertension and dizziness. Our results indicated that the odds ratio is 1.30 for hypertension and 1.26 for Dizziness than those without such comorbidities. Higher prevalence of hypertension may be one reason. A Taiwanese research showed that higher crude prevalence of hypertension was found in the elderly population [23].

Pharmaceutical care services are an important part of medication safety. According to the results of the GEE model analysis, the average number of outpatient visits during the following year for subjects participating in the plan significantly increased for 0.117 visits. It may be due to that Pharmaceutical Home Care Plan has a priority order for the counseling cases. In medical expenses, pharmacist intervention may improve the medication behavior of patients with frequent visits, showing the effect of counseling. The intervention of community pharmaceutical care could enhance compliance with medications of the elderly, prevent the occurrence of serious diseases, and could save medical costs [24,25]. A systematic literature review indicated that the clinical benefits of hospital pharmaceutical services include the improvement of patients’ health status, reduction of the use of undesirable drugs, and meeting the cost of Quality-Adjusted Life Years (QALYs), or cost savings [26]. It was found that the average outpatient medical expenses for patients with frequent visits in the plan were significantly lower than that of non-participants for 18,302 points. It may be that the intervention of pharmacist’s consultation resulted in an improvement in medication for patients with frequent visits, thereby reducing medical expenses. In this study, the average number of outpatient visits and medical expenses was significantly reduced among patients joining the pharmaceutical home care plan. Although compared with patients without joining the pharmaceutical home care plan, the average number of physician visits was still higher on statistical significant meaning, the difference is very limited. In terms of outpatient medical expenses, it was dramatically decreased after participating in the pharmaceutical home care. It showed the pharmaceutical home care helped reduce medical service utilization. The outpatient visits were not decreased as it may be related to the aging society, due to the complex health conditions of high-need patients suffering from chronic diseases, as well as the number of medications prescribed that are polypharmacy. It may require long-term intervention and counseling by a pharmacist service to improve high-frequency behaviors.

After joining the experimental plan of pharmaceutical home care, the average number of outpatient visits was similar and the medical expense was lower when compared with those who did not participate in the plan. The primary reason for the effectiveness of the Pharmaceutical Home Care Pilot Program is the virtuous cycle given by the policy. For reducing the waste of medical resources, the National Health Insurance Administration appoints community pharmacists to perform the home medication care, and pay for the pharmacy care fees. The community pharmacists would have face-to-face communication with high frequent users. While most high frequent users are the elderly or patients with multiple chronic diseases, the direct consultation and continuous instruction provided by community pharmacists to them, to establish a guideline for medication to reduce duplicate waste of medications, indirectly reduces the number of physician visits and achieves reasonable utilization of the medical resource. The government, community pharmacists, and patients are all beneficiaries of the policy. The virtuous cycle is an important factor contributing to a win-win policy. The pharmaceutical home care program continues to this day. It indicated that most patients agreed pharmaceutical service is helpful, they are highly satisfied, and wished to continue. Pharmacists can resolve drug therapy problems with a specifically trained skill in this plan. In terms of economic outcomes, the outpatient drug expenditure decreased. The effectiveness of counseling can be sustained after the completion of the plan, which is worth having a further follow-up study to provide more information for planning policies of medical and health-related units. Moreover, the Ministry of Health and Welfare Taiwan (MOHW) integrated the pharmaceutical home care program and other pharmaceutical programs into the “Special Population Pharmaceutical Care Program” in 2019 for patients with high-frequent visits, new residents, migrant workers, people with disabilities, and the aboriginal in Taiwan.

Compared with the previous studies, the study not only evaluated the impact of participation on medical utilization but also investigated the influence factors related to the willingness of high frequent users of outpatient services to participate in the plan of pharmaceutical home care. Based on the results of this study, the effectiveness of the program has been shown and verified. The major assignment of the next stage is how to increase the participation rate of the program. The research findings would provide a reference for participation improvement of the present Pharmaceutical Care Program.

There may be some possible limitations in this study. This study uses the secondary data analysis of the NHI Database. The database does not contain information on the quality of life, education, attitudes, and living habits. Therefore, we cannot understand the difference in the quality of life and the improvement of knowledge and attitudes in home-based medical care. Only the analysis of health insurance medical utilization may underestimate the impact and effectiveness of the pharmacist’s intervention and counseling on patients with frequent visits. In addition, the patients who participated in the Pharmaceutical Home Care Pilot Program were voluntary and the dates of joining and leaving the programs of each patient were different. It may have selection bias in the study. The study reduced the selection bias by using the nationwide database and selecting the patients without any exclusion, whereas the highly frequent users of medical resources are in the minority. As the number of study subjects was rare, our study was unable to use matching methods to further reduce the bias. Future research can use the matching methods to obtain study subjects to further assess the benefits of pharmaceutical home care. Due to the LHID limitation, the study used the 2010–2013 database to assess the impact of pharmaceutical home care on medical utilization. This study was based on a nationwide database. Thus, the study subjects and results still had accuracy and representativeness.

The research subjects cannot exclude the high-visit insurance subjects of the other integrated care plan. It is possible that patients with frequent visit in the control group joined the integrated care plan, and maybe not truly measure the high-visit illnesses of the pharmaceutical home care program and the difference between the frequent patients who have not joined any plan. The literature points out that patients with mental disorders are more likely to become patients with frequent visits, but the design of this medical home care pilot plan excludes the number of visits to the psychiatric department. Therefore, mental illness is not included as a comorbidity in this study, which may cause differences in the results of this study and other high-visit studies.

## 5. Conclusions

Based on the results of this study, the effectiveness of the program has been shown and verified. After joining the Pharmaceutical Home Care Pilot Program, the average number of outpatient visits decreased significantly and the medical expense was lower when compared with those who did not participate in the plan.

## Figures and Tables

**Table 1:** The baseline characteristics of patients with a high frequency of visits.

Variable	Join Pharmaceutical Home Care Plan						
	Total		No		Yes		*p* Value
	*N*	%	*N*	%	*N*	%	
Total	3943	100.00	3352	85.01	591	14.99	
Gender							<0.001
Female	1921	48.72	1630	84.85	291	15.15	
Male	2022	51.28	1722	85.16	300	14.84	
Age (year)							<0.001
<44	314	7.96	282	89.81	32	10.19	
45–55	432	10.96	379	87.73	53	12.27	
55–65	721	18.29	600	83.22	121	16.78	
65–75	1108	28.10	919	82.94	189	17.06	
75–85	1127	28.58	951	84.38	176	15.62	
>85	241	6.11	221	91.70	20	8.30	
Degree of urbanization							<0.001
High	2742	69.54	2381	86.83	361	13.17	
Middle	745	18.89	610	81.88	135	18.12	
Low	456	11.56	361	79.17	95	20.83	
CCI score							0.004
0	422	10.70	373	88.39	49	11.61	
1–2	1294	32.82	1067	82.46	227	17.54	
3–4	1086	27.54	921	84.81	165	15.19	
>5	1141	28.94	991	86.85	150	13.15	
Hypertension							<0.001
No	1419	35.99	1243	87.60	176	12.40	
Yes	2524	64.01	2109	83.56	415	16.44	
Diabetes							<0.001
No	2415	61.25	2049	84.84	366	15.16	
Yes	1528	38.75	1303	85.27	225	14.73	
Allergic rhinitis							0.661
No	3338	84.66	2835	84.93	503	15.07	
Yes	605	15.34	517	85.45	88	14.55	
Musculoskeletal system and connective tissue diseases							<0.001
No	650	16.48	573	88.15	77	11.85	
Yes	3293	83.52	2779	84.39	514	15.61	
Digestive system diseases							<0.001
No	625	15.85	548	87.68	77	12.32	
Yes	3318	84.15	2804	84.51	514	15.49	
Sleep disturbance							<0.001
No	2790	70.76	2385	85.48	405	14.52	
Yes	1153	29.24	967	83.87	186	16.13	
Dizziness							<0.001
No	2268	57.52	1970	86.86	298	13.14	
Yes	1675	42.48	1382	82.51	293	17.49	

**Table 2:** Factors related to the willingness of high frequent users of outpatient services to participate in the plan of pharmaceutical home care.

Variable	Adjusted Odds Ratio	95% CI		*p* Value
		Lower	Upper	
Gender				
Female (ref.)				
Male	1.06	0.86	1.30	0.593
Age (year)				
<44 (ref.)				
45–55	1.06	0.64	1.78	0.815
55–65	1.45	0.90	2.31	0.125
65–75	1.41	0.89	2.23	0.142
75–85	1.20	0.75	1.92	0.437
>85	0.69	0.36	1.31	0.259
Degree of urbanization				
High (ref.)				
Middle	1.41	1.11	1.80	0.005
Low	1.66	1.26	2.19	<0.001
CCI score				
0 (ref.)				
1–2	1.42	0.95	2.11	0.087
3–4	1.26	0.84	1.90	0.269
>5	1.14	0.75	1.74	0.532
Hypertension				
No (ref.)				
Yes	1.30	1.04	1.62	0.022
Diabetes				
No (ref.)				
Yes	0.90	0.73	1.11	0.313
Allergic rhinitis				
No (ref.)				
Yes	1.04	0.79	1.38	0.761
Musculoskeletal system and connective tissue diseases				
No (ref.)				
Yes	1.19	0.89	1.58	0.243
Digestive system diseases				
No (ref.)				
Yes	1.30	0.97	1.73	0.077
Sleep disturbance				
No (ref.)				
Yes	1.12	0.91	1.38	0.288
Dizziness				
No (ref.)				
Yes	1.26	1.04	1.54	0.021

**Table 3:** The impact of participation in the program on the number of outpatient visits.

Variable	Average Number of Outpatient Visits						
	Mean	Std	β	S.E.	95% CI		*p* Value
Total	94.64	40.31					
Pharmaceutical home care plan							
No (ref.)	92.78	40.22					
Yes	105.35	38.84	0.117	0.02	0.08	0.15	<0.001
Gender							
Female (ref.)	95.15	37.12					
Male	94.14	43.05	0.011	0.02	−0.02	0.04	0.514
Age (year)							
<44 (ref.)	93.51	44.94					
45–55	100.85	52.27	0.059	0.04	−0.01	0.13	0.118
55–65	96.43	41.49	−0.006	0.04	−0.08	0.06	0.862
65–75	95.52	36.07	−0.019	0.03	−0.08	0.05	0.560
75–85	92.50	36.24	−0.052	0.03	−0.12	0.01	0.128
>85	85.68	36.45	−0.118	0.04	−0.20	−0.03	0.007
Degree of urbanization							
High (ref.)	95.12	41.19					
Middle	92.36	38.22	−0.048	0.02	−0.09	−0.01	0.018
Low	95.82	38.13	−0.016	0.02	−0.06	0.03	0.525
CCI score							
0 (ref.)	92.47	39.33					
1–2	95.85	36.50	0.022	0.02	−0.02	0.07	0.335
3–4	96.72	41.01	0.038	0.03	−0.02	0.09	0.161
>5	92.17	43.47	−0.002	0.03	−0.06	0.05	0.956
Hypertension							
No (ref.)	94.61	44.04					
Yes	94.65	38.02	−0.002	0.02	−0.04	0.03	0.928
Diabetes							
No (ref.)	94.77	39.99					
Yes	94.44	40.76	0.001	0.02	−0.03	0.03	0.931
Allergic rhinitis							
No (ref.)	94.43	40.86					
Yes	95.69	36.85	0.008	0.02	−0.03	0.05	0.699
Musculoskeletal system and connective tissue diseases							
No (ref.)	85.24	38.79					
Yes	96.50	40.35	0.121	0.02	0.08	0.17	<0.001
Digestive system diseases							
No (ref.)	90.23	38.19					
Yes	95.47	40.64	0.042	0.02	0.00	0.08	0.038
Sleep disturbance							
No (ref.)	93.51	39.18					
Yes	97.34	42.40	0.024	0.02	−0.01	0.06	0.160
Dizziness							
No (ref.)	93.03	40.61					
Yes	96.81	39.81	0.032	0.02	0.00	0.06	0.048

Abbreviations: Std, standard deviation; S.E., standard error.

**Table 4:** The impact of participation in the pharmaceutical home care plan on outpatient medical expenses.

Variable	Average Outpatient Medical Expenses ^1^						
	Mean	Std	β	S.E.	95% CI		*p* Value
Total	101,915	153,685					
Pharmaceutical home care plan							
No (ref.)	105,725	163,814					
Yes	81,269	66,629	−18,302	4081	−26,301	−10,303	<0.001
Gender							
Female (ref.)	98,469	133,680					
Male	105,207	170,215	2098	6555	−10,749	14,945	0.749
Age (year)							
<44 (ref.)	101,903	289,451					
45–55	91,466	118,995	−14,952	25,547	−65,024	35,120	0.558
55–65	120,412	177,452	−348	24,762	−48,881	48,184	0.989
65–75	104,636	139,895	−18,714	23,565	−64,900	27,472	0.427
75–85	93,281	105,416	−35,486	23,958	−82,443	11,471	0.139
>85	92,440	107,435	−48,148	25,613	−98,348	2053	0.060
Degree of urbanization							
High (ref.)	108,377	168,909					
Middle	89,278	115,301	−13,914	5956	−25,588	−2240	0.020
Low	83,720	100,588	−21,087	6843	−34,498	−7676	0.002
CCI score							
0 (ref.)	63,450	164,988					
1–2	70,602	92,185	19,785	5911	8199	31,372	0.001
3–4	92,014	111,174	44,913	8416	28,417	61,409	<0.001
>5	161,201	200,767	113,835	11,248	91,790	135,881	<0.001
Hypertension							
No (ref.)	101,304	188,322					
Yes	102,173	129,999	804	6122	−11,195	12,803	0.896
Diabetes							
No (ref.)	92,567	155,144					
Yes	116,742	150,310	−2039	5861	−13,525	9448	0.728
Allergic rhinitis							
No (ref.)	102,659	153,907					
Yes	97,567	140,140	−7555	6333	−19,967	4858	0.233
Musculoskeletal system and connective tissue diseases							
No (ref.)	113,522	238,417					
Yes	99,809	130,617	−11,347	10,847	−32,606	9912	0.296
Digestive system diseases							
No (ref.)	101,368	195,784					
Yes	102,028	143,689	−4490	7319	−18,834	9854	0.540
Sleep disturbance							
No (ref.)	103,632	158,937					
Yes	97,825	140,354	−3640	6395	−16,173	8894	0.569
Dizziness							
No (ref.)	112,287	178,967					
Yes	87,866	109,098	−18,045	5009	−27,862	−8228	<0.001

^1^ The unit of medical expense claim was the “point”. One point was approximately equal to 0.03 US dollars. Abbreviations: Std, standard deviation; S.E., standard error.

**Table 5:** The differences in medical utilization among patients participating in the Pharmaceutical Home Care Pilot Program (*N* = 591).

Variables	Before Join	After Join	Differences	*p* Value
Average outpatient visits	111.98	105.35	6.63	<0.001
Average outpatient expenses ^1^	91,140	81,269	9871	<0.001

^1^ The unit of medical expense claim was the “point”. One point was approximately equal to 0.03 US dollars.

## Data Availability

The data that support the findings of this study are available from the corresponding author, upon reasonable request.
